# AI-Powered Identification of Osteoporosis in Dental Panoramic Radiographs: Addressing Methodological Flaws in Current Research

**DOI:** 10.3390/diagnostics14202298

**Published:** 2024-10-16

**Authors:** Robert Gaudin, Shankeeth Vinayahalingam, Niels van Nistelrooij, Iman Ghanad, Wolfus Otto, Stephan Kewenig, Carsten Rendenbach, Vasilios Alevizakos, Pascal Grün, Florian Kofler, Max Heiland, Constantin von See

**Affiliations:** 1 Department of Oral and Maxillofacial Surgery, Charité—Universitätsmedizin Berlin, Corporate Member of Freie Universität Berlin and Humboldt Universität zu Berlin, Augustenburger Platz 1, 13353 Berlin, Germany; robert-andre.gaudin@charite.de (R.G.); niels.vannistelrooij@radboudumc.nl (N.v.N.); iman.ghanad@charite.de (I.G.); wolfus.otto@charite.de (W.O.); stephan.kewenig@charite.de (S.K.); carsten.rendenbach@charite.de (C.R.); max.heiland@chairte.de (M.H.); 2 Berlin Institute of Health at Charité—Universitätsmedizin Berlin, 10178 Berlin, Germany; 3 Department of Oral and Maxillofacial Surgery, Radboud University Medical Center, 6500 HB Nijmegen, The Netherlands; shankeeth.vinayahalingam@radboudumc.nl; 4 Research Centre for Digital Technologies in Dentistry and CAD/CAM, Danube Private University, 3500 Krems an der Donau, Austria; constantin.see@dp-uni.ac.at; 5 Center for Oral and Maxillofacial Surgery, Faculty of Medicine/Dental Medicine, Danube Private University, 3500 Krems an der Donau, Austria; pascal.gruen@charite.de; 6 Helmholtz AI, Helmholtz Zentrum München, Ingostaedter Landstrasse 1, 85764 Oberschleissheim, Germany; florian.kofler@tum.de; 7 TUM-NeuroImaging Center, Klinikum Rechts der Isar, Technical University of Munich, 81675 Munich, Germany

**Keywords:** osteoporosis detection, panoramic radiographs, AI application, deep learning, EfficientNet classification

## Abstract

**Background:** Osteoporosis, a systemic skeletal disorder, is expected to affect 60% of women over 50. While dual-energy X-ray absorptiometry (DXA) scans are the current gold standard for diagnosis, they are typically used only after fractures occur, highlighting the need for early detection tools. Initial studies have shown panoramic radiographs (PRs) to be a potential medium, but these have methodological flaws. This study aims to address these shortcomings by developing a robust AI application for accurate osteoporosis identification in PRs. **Methods:** A total of 348 PRs were used for development, 58 PRs for validation, and 51 PRs for hold-out testing. Initially, the YOLOv8 object detection model was employed to predict the regions of interest. Subsequently, the predicted regions of interest were extracted from the PRs and processed by the EfficientNet classification model. **Results:** The model for osteoporosis detection on a PR achieved an overall sensitivity of 0.83 and an F1-score of 0.53. The area under the curve (AUC) was 0.76. The lowest detection sensitivity was for the cropped angulus region (0.66), while the highest sensitivity was for the cropped mental foramen region (0.80). **Conclusion:** This research presents a proof-of-concept algorithm showing the potential of deep learning to identify osteoporosis in dental radiographs. Furthermore, our thorough evaluation of existing algorithms revealed that many optimistic outcomes lack credibility when subjected to rigorous methodological scrutiny.

## 1. Introduction

In recent years, the field of dentistry has undergone a remarkable evolution, spurred by the integration of cutting-edge technologies such as intraoral scanners, 3D printers, and artificial intelligence (AI) imaging applications [1,2,3]. These advancements have significantly enhanced the automation of standardized dental procedures. Traditionally, dental diagnoses and treatment planning have relied heavily on the subjective judgment and expertise of dental professionals, introducing variability into clinical outcomes. Unlike other medical fields, dentistry, alongside radiology, uniquely incorporates AI in the routine analysis and classification of radiographic images in technologically advanced practices [4,5,6,7].

Osteoporosis is a systemic condition characterized by reduced bone mass and structural deterioration, which not only predisposes individuals to fractures but also adversely affects oral health, particularly impacting the integrity of the alveolar bone [8,9]. Furthermore, this can complicate dental interventions, such as the placement of implants, mandibular and alveolar fracture healing, bone augmentation, and periodontal procedures [10].

The current gold standard for osteoporosis detection is the dual-energy X-ray absorptiometry (DXA) scan, which is only present in specialized osteoporosis centers and is typically employed post-fracture [11,12]. Patients exhibiting a bone mineral density (BMD) T-score above −1 are classified as healthy, while those with a BMD T-score below −2.5 are diagnosed with osteoporosis [13]. Consequently, the interpretation of cross-sectional investigations and individual longitudinal diagnoses necessitates meticulous consideration of the unique circumstances surrounding each case. Generalizations made within mathematical models are subject to clinical controversy and warrant thorough discussion [14]. The increased burden on the healthcare system is in double-digit billions of dollars worldwide; osteoporosis-related fractures and osteoporosis-related procedures critically demand tools capable of early detection [15].

The panoramic radiography (PR) is a routine component of dental evaluations and gives a 2D overview of the dentition, the jaw, and the hard surrounding tissue [16,17]. This may be conducted annually and presents a viable medium for early detection efforts. PR provides a comprehensive view of the dentition, maxillary sinuses, and mandibular joints and is instrumental in assessing changes in mandibular cortical width and trabecular patterns indicative of osteoporosis [18,19,20,21]. In this context, specific measures derivable from dental PRs have been developed to assist in osteoporosis identification [22,23,24,25]. The Klemetti Index (KI), introduced by Klemetti in 1993, calculates the mandibular cortical index and has since become the most frequently utilized index for this purpose [20,26]. Nevertheless, these indices are rarely used in the clinical workflow, since they must be manually calculated, are prone to errors, and are time-consuming. Comparing these to a gold-standard technique like DXA warrants fundamental enquiry [27,28].

Recent studies utilizing deep convolutional neural networks for the analysis of PR images have reported promising results in detecting osteoporotic changes [11,29,30]. However, these studies show critical flows by overlooking critical variables such as age, gender, disease severity, and radiographic technique, which can influence the accuracy of osteoporosis detection. This oversight underscores the importance of a nuanced approach to interpreting AI-driven findings [4,5].

By exploring the mathematical complexities involved in implementing these technologies, this study aims to address these aforementioned flaws and develop a robust proof-of-concept image classifier to detect osteoporosis in PRs.

Furthermore, the aim is to shed light on the thorough approach of developing such an algorithm and advocate for more refined open-source AI methodologies, thereby enhancing diagnostic accuracy and patient care.

## 2. Materials and Methods

The current study was conducted according to the guidelines of the Medical World Association (Declaration of Helsinki). Ethical approval was granted by the Institutional Review Board, Charité ethics committee (EA2/184/23). The checklist for AI research in dentistry of the ITU/WHO focus group “Artificial Intelligence for Health (AI4H)” was consulted for the reporting in this study [4].

### 2.1. Data

The study retrospectively included patients with a clinical suspicion of osteoporosis from Danube Private University. Each patient underwent a DXA scan to assess their BMD. Patients exhibiting a BMD T-score above −1 were classified as healthy, while those with a BMD T-score below −2.5 were diagnosed with osteoporosis. PRs were retrieved from the medical records of each patient after the DXA scan.

### 2.2. Data Preparation

**Figure 1:**
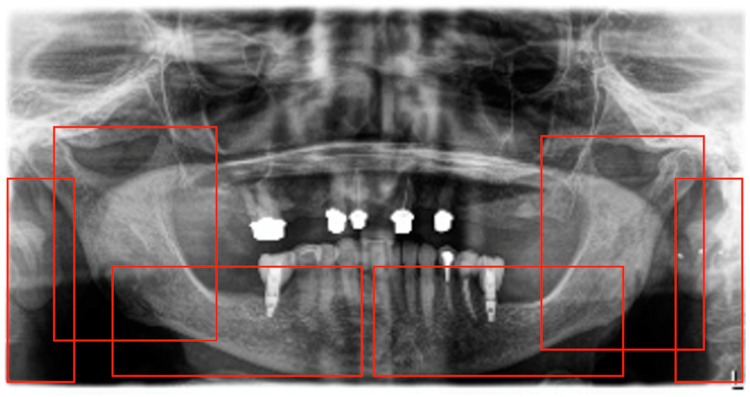
In each panoramic X-ray (PR), six regions of interest were identified: the left and right projections of the vertebral spine, the left and right posterior regions of the mandible (including the angulus, ramus, condyle, and coronoid process), and the left and right anterior regions of the mandible, extending from the symphysis up to the angulus.

In each PR, six regions of interest were identified. These regions comprised the left and right projections of the vertebral spine and the left and right posterior regions of the mandible, including the angulus, ramus, condyle, and coronoid process. Additionally, the left and right anterior regions of the mandible, extending from the symphysis up to the angulus, were included (Figure 1). Each discerned region was annotated with a bounding box and categorized as vertebral, angulus, or foramen.

The PRs underwent stratification based on the osteoporosis diagnosis, resulting in the allocation of 348 PRs for training, 58 PRs for validation, and 51 PRs for hold-out testing. All PRs belonging to a specific patient were exclusively used for either training, validation, or testing purposes. The training set comprised vertebral, angulus, and foramen regions. Similarly, the validation set consisted of 34 vertebral, 62 angulus, and 55 foramen regions, while the test set included 34 vertebral, 62 angulus, and 55 foramen regions.

### 2.3. Model

A two-stage deep learning model was formulated for osteoporosis detection. Initially, the YOLOv8 object detection model was employed to predict the regions of interest. Subsequently, the predicted regions of interest were extracted from the PR and subjected to processing by the EfficientNet classification model. This classification model was tasked with categorizing each region as either indicative of osteoporosis or not.

The classification stage was repurposed for different regions, allowing an exploration of the model’s effectiveness in detecting osteoporosis across various anatomical areas. Based on the knowledge of prior research, it was anticipated that the dentition would have a confounding effect. Therefore, specifically, three distinct configurations of the classification stage were devised for (A) the vertebral region, (B) the angulus region, and (C) the foramen region.

### 2.4. Model Training

The region detection stage was implemented utilizing MMYOLO (v.0.6.0), based on PyTorch (v.2.0.1) [31,32]. Model parameters pre-trained on the COCO dataset were utilized for the model initialization [33]. Stochastic gradient descent (SGD) served as the optimizer, incorporating a weight decay of 0.0005. The initial learning rate of 0.01 underwent linear reduction during training, spanning a maximum of 500 epochs.

For the classification stage, MMPreTrain (v.1.0.1) based on PyTorch (v.2.0.1) was employed [32,34]. A region of interest underwent mean-padding to achieve a square aspect ratio before undergoing processing by a classifier. Pretraining utilized the ImageNet dataset, and the AdamW optimizer was applied with a weight decay of 0.05 [35,36]. The learning rate experienced a linear warm-up to 0.001, followed by a cosine annealing schedule, extending up to a maximum of 60 epochs. Both training and inference operations were conducted on a workstation featuring a 48 GB GPU and 128 GB memory (RTX A6000, NVIDIA, Santa Clara, CA, USA).

### 2.5. Model Evaluation

The accuracy of osteoporosis detection in the vertebral, angulus, and foramen regions involved the processing of each cropped region through its corresponding classifiers. Specifically, the osteoporosis probability derived from both the region-specific classifier and the region-agnostic classifier, along with the average probability, were compared against the gold-standard osteoporosis category.

Based on these predictions, performance metrics were computed, including precision (Precision = TP/(TP + FP)), sensitivity (Sensitivity = TP/(TP + FN)), specificity (Specificity = TN/(TN + FP)), accuracy (Accuracy = (TP + TN)/(TP + TN + FP + FN)), and F1-score (F1-score = 2 TP/(2 TP + FP + FN)), where TP, TN, FP, and FN represent true positive, true negative, false positive, and false negative, respectively.

Furthermore, a confusion matrix was presented for each region, along with a receiver operating characteristic (ROC) curve, from which the AUC was computed.

## 3. Results

**Figure 2:**
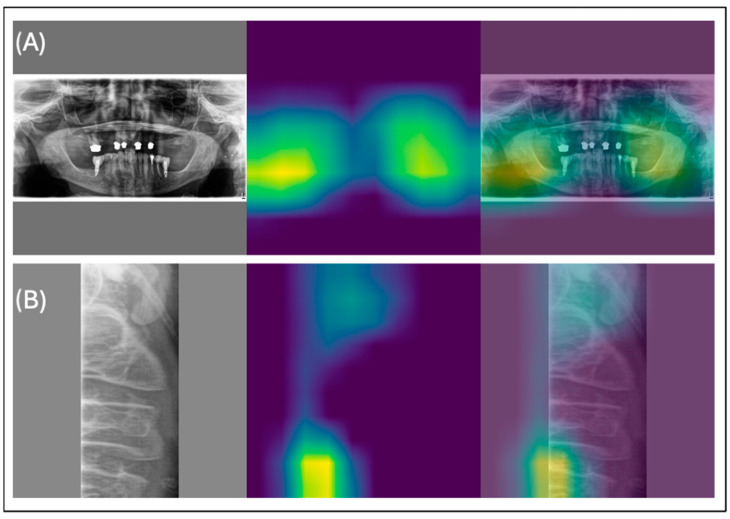
Examples of true positive predictions are shown in (**A**) for osteoporosis detection in a panoramic radiograph with no cropped region of interest (ROI) and (**B**) for osteoporosis detection in a panoramic X-ray (PR) with the cropped vertebra.

**Figure 3:**
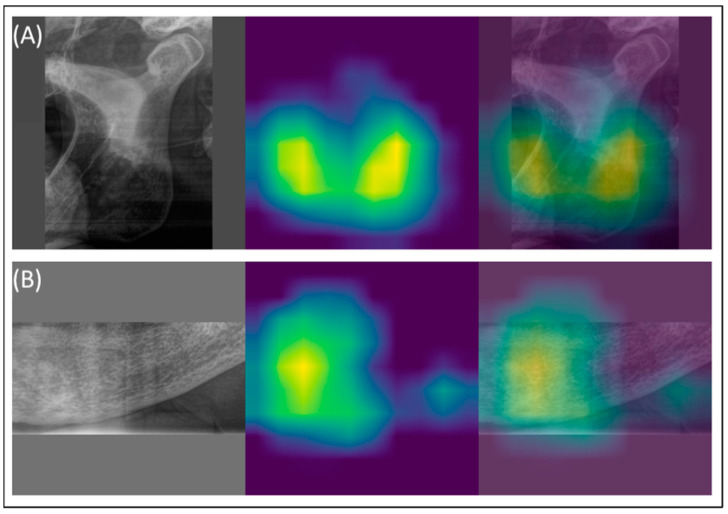
Examples of true positive predictions are shown in (**A**) for osteoporosis detection in a panoramic radiograph with the cropped angulus and (**B**) for osteoporosis detection in a panoramic radiograph with the cropped mental foramen.

The model, when applied to panoramic radiographs (PR) without cropping to specific ROIs, achieved an overall sensitivity of 0.83, an F1-score of 0.53, and an AUC of 0.64. Examples of true positive predictions are shown in Figure 2 and Figure 3. Table 1 summarizes the detection performance for each ROI (vertebra, angulus, and foramen) based on the test set, where each cropped region was processed through its corresponding classifier.

**Figure 4:**
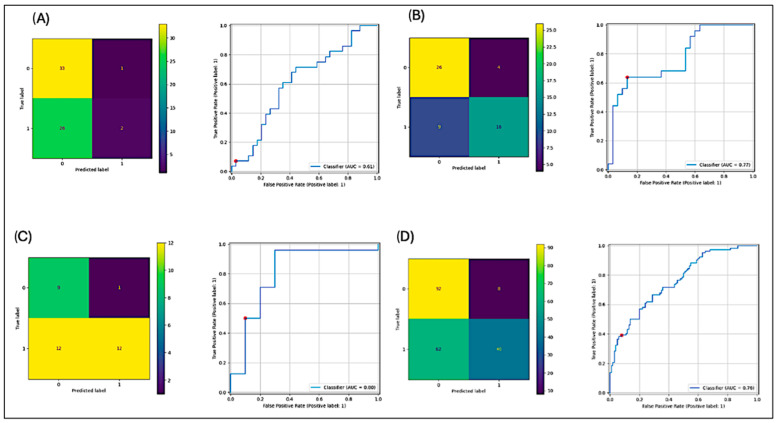
(**A**) Angulus, (**B**) foramen, (**C**) vertebra, and (**D**) all.

The ROC curves and confusion matrices for the angulus, foramen, and vertebra offer a detailed assessment of the model’s performance in these specific anatomical regions (Figure 4).

The ROC curves highlight the angulus as the most challenging region, with the lowest detection sensitivity of 0.66 (two out of three cases). In contrast, the vertebra achieved the highest sensitivity at 0.92 (12 out of 13 cases). The foramen showed promising performance as well, with a sensitivity of 0.80 (16 out of 20 cases).

The confusion matrices further support these findings. The angulus region shows a higher rate of false negatives, indicating that osteoporotic cases in this area are more likely to be misclassified. Conversely, the vertebra demonstrates more balanced classification results, with fewer false positives and false negatives, reflecting its higher diagnostic accuracy.

## 4. Discussion

PR, commonly utilized in dental evaluations, provides a comprehensive view that is potentially useful for early osteoporosis detection. Despite the development of specific measures like the Klemetti Index to assist in identifying osteoporosis from these radiographs, such indices are infrequently used due to their manual, error-prone, and time-consuming nature [20].

Recent advancements in AI, particularly deep convolutional neural networks, have shown promise in analyzing PR to detect osteoporotic changes [11,30]. However, these studies often fail to account for variables such as age, gender, disease severity, and radiographic technique, which significantly affect diagnostic accuracy.

This study explored the optimal use of a deep learning algorithm to detect osteoporosis from dental radiographs, achieving a proof-of-concept algorithm with an overall sensitivity of 0.83. To define the best region of interest initially, the YOLOv8 object detection model was employed. Notably, the regions with the highest bony structure yielded the most promising results, with sensitivities of 0.92 for the vertebra and 0.80 for the foramen of the mandible. These regions of interest are parameters in established osteoporosis indices such as the KI and DXA analysis [20,23].

The variations in sensitivity across different anatomical regions, particularly the vertebra (0.92), foramen of the mandible (0.80), and angulus of the mandible (0.66), can be attributed to several anatomical and radiographic factors that influence the detection of osteoporotic changes.

Firstly, the anatomical characteristics of these regions play a significant role. The vertebra consists of a dense network of trabecular bone that is especially sensitive to changes due to osteoporosis. The unique architecture of trabecular bone allows for more pronounced alterations in bone density, making it easier for the algorithm to identify these changes during image analysis. In contrast, the angulus of the mandible has a more complex geometry and a higher proportion of cortical bone, which can obscure subtle variations in bone density associated with osteoporosis. This region also exhibits thinner bone areas, resulting in less distinctive radiographic features compared to the vertebral bodies.

Secondly, radiographic technique and projection are critical in determining sensitivity. The vertebrae are typically imaged using standard positions that optimize the visualization of bony structures, contributing to clearer images with reduced superimposition of surrounding tissues. This is particularly true for the lumbar spine, which is frequently targeted in osteoporosis assessments. Conversely, the angulus of the mandible can be influenced by patient positioning and the angulation of the X-ray beam, potentially leading to less clear images. Additionally, variations in mandibular morphology across patients may complicate the detection of osteoporotic changes in the angulus region.

The aim of our study is to preferably recognize all true positives and therein create an imbalance between false and true positives with respect to false positives. Therefore, the sensitivity is high, and F1 score, which is calculated with the false positive patients in this study, has a low performance, ranging from 0.12 to 0.71 (Table 1). This is a typical bias factor in binary classification systems with the goal of developing algorithms for preventive detection of pathologies, in this case osteoporosis.

The osteoporosis detection model achieved an overall sensitivity of 0.83, an F1-score of 0.53, and an AUC of 0.64 when analyzing panoramic radiographs (PR) without cropped regions of interest (ROIs). True positive predictions are illustrated in Figure 2 and Figure 3, while Table 1 summarizes detection performance for specific ROIs in the vertebral, angulus, and foramen regions. The confusion matrices in Figure 4 show F1 scores ranging from 0.12 to 0.71 for these cropped regions.

The angulus region had the lowest sensitivity (0.66; 2 out of 3), while the vertebral region achieved the highest (0.92; 12 out of 13). Notably, the foramen of the mandible showed promise for osteoporosis detection, with a sensitivity of 0.80 (16 out of 20). This pattern aligns with established clinical practice, where the vertebra is frequently the primary focus for osteoporosis diagnosis. Vertebral fractures are common indicators of osteoporosis, and diagnostic tools and imaging techniques are often optimized for this region, explaining the model’s superior performance in the vertebra [37].

On the other hand, the angulus, due to its anatomical complexity and variability, presents more challenges for both clinical diagnosis and model classification. This variability contributes to the model’s lower sensitivity and F1-score in this region.

The lower F1-score in regions like the angulus can be attributed to anatomical complexity, insufficient training data, poor image quality, and ineffective feature extraction. To improve performance, strategies such as augmenting training data, advanced feature engineering, model fine-tuning, handling class imbalance, utilizing ensemble methods, and applying post-processing techniques can be implemented.

By addressing these factors and adopting these improvements, the model’s effectiveness in detecting osteoporosis across various anatomical areas can be enhanced, ultimately leading to better clinical outcomes and improved patient care.

It is important to address that, for technical reasons, the regions of interest, particularly the spine, can appear with varying levels of sharpness in PRs. This variability in image quality can significantly influence the accuracy of AI-assisted osteoporosis diagnosis. Differences in sharpness can result from a variety of factors, including the positioning of the patient, the quality of the imaging equipment, and the settings used during the radiograph capture. These inconsistencies can lead to challenges in data annotation and analysis, as the AI algorithms may struggle to accurately detect and assess osteoporotic changes in images that are not uniformly clear. Consequently, ensuring high and consistent image quality is crucial for the reliability of AI diagnostics, as highlighted by the comprehensive analyses of Lee et al. and Sukegawa et al. [11,30]. The disparities in image sharpness underscore the need for standardized imaging protocols and advanced preprocessing techniques to enhance the performance and robustness of AI systems in medical diagnostics.

Notably, incorporating younger patients into the dataset has proven to have a misleading positive effect on the algorithm’s performance, as it merely differentiates between young and old. Furthermore, including data from another X-ray manufacturer has shown to have the same effect.

Despite the promising results for a proof of concept, this study has several limitations. First, all data were collected from one medical center, resulting in a database consisting of the local population. Additionally, the PRs were acquired using only one imaging device. The model’s performance may degrade when implemented with a different device, as this study did not account for clinical settings in which PRs may be acquired with different devices. Furthermore, the algorithm was employed only within the domain of the training and test sets. Further investigations are necessary to validate the effectiveness of this architecture for other clinical tasks and real-world scenarios. More data and extending the labeled data with PR from multiple medical centers and imaging devices is necessary to improve the model’s robustness and generalizability. Lastly, the diagnostic accuracy of the proposed model should be evaluated in a prospective clinical setting [38].

## 5. Conclusions

In conclusion, while the study highlights the potential of AI in transforming early osteoporosis screening, it also acknowledges several limitations that necessitate further investigation to validate the results and enhance diagnostic accuracy. Addressing these limitations is crucial for building a robust evidence base that supports the findings. Ultimately, a broader and more informed application of technology in medical imaging can lead to more accurate diagnoses and improved patient outcomes in the future.

## Figures and Tables

**Table 1:** Detection performance (osteoporosis/non-osteoporosis) of specific regions of interest (ROIs) in the vertebral, angulus, and foramen regions, which involved the processing of each cropped region.

	Angulus	Foramen	Vertebral	All	
Measure	Value	Value	Value	Value	Derivations
Sensitivity	0.6667	0.8000	0.9231	0.8333	TPR = TP/(TP + FN)
Specificity	0.5593	0.7429	0.4286	0.5974	SPC = TN/(TP + TN)
Precision	0.0714	0.6400	0.5000	0.3922	PPV = TP/(TP + FP)
Negative Predictive Value	0.9706	0.8667	0.9000	0.9200	NPV = TN/(TN + FN)
False Positive Rate	0.4407	0.2571	0.5714	0.4026	FPR = FP/(TP + TN)
False Discovery Rate	0.9286	0.3600	0.5000	0.6078	FDR = FP/(FP + TP)
False Negative Rate	0.3333	0.2000	0.1667	0.1667	FNR = FN/(FN + TP)
Accuracy	0.5645	0.7636	0.6176	0.6535	ACC = (TP + TN)/(P + N)
F1 Score	0.1290	0.7111	0.6486	0.5333	F1 = 2TP/(2TP + FP + FN)
Matthews Correlation Coefficient	0.0974	0.5244	0.3750	0.3667	MCC = TP*TN − FP*FN/sqrt((TP + FP)*(TP + FN)*(TN + FP)*(TN + FN))
AUC (Area under the curve)	0.61	0.77	0.80	0.76	

## Data Availability

The data that support the findings of this study are available on request from the corresponding author, [VA]. The data are not publicly available due to their containing information that could compromise the privacy of research participants.

## References

[1] Schlenz M.A., Schulz-Weidner N., Olbrich M., Buchmann D., Wöstmann B. (2023). Insights on the digitalisation of dental practices: A cross-sectional pilot study in Hesse. Int. J. Comput. Dent..

[2] Shen Z., Song L. (2020). Trend of collaboration and sharing in digitalized restorative dentistry. Chin. J. Pract. Stomatol..

[3] Gross D., Gross K., Wilhelmy S. (2019). Digitalization in dentistry: Ethical challenges and implications. Quintessence Int..

[4] Schwendicke F., Singh T., Lee J.H., Gaudin R., Chaurasia A., Wiegand T., Uribe S., Krois J., on behalf of the IADR e-oral health network and the ITU WHO focus group AI for Health (2021). Artificial intelligence in dental research: Checklist for authors, reviewers, readers. J. Dent..

[5] Gichoya J.W., Thomas K., Celi L.A., Safdar N., Banerjee I., Banja J.D., Seyyed-Kalantari L., Trivedi H., Purkayastha S. (2023). AI pitfalls and what not to do: Mitigating bias in AI. Br. J. Radiol..

[6] Mörch C.M., Atsu S., Cai W., Li X., Madathil S.A., Liu X., Mai V., Tamimi F., Dilhac M.A., Ducret M. (2021). Artificial Intelligence and Ethics in Dentistry: A Scoping Review. J. Dent. Res..

[7] Endres M.G., Hillen F., Salloumis M., Sedaghat A.R., Niehues S.M., Quatela O., Hanken H., Smeets R., Beck-Broichsitter B., Rendenbach C. (2020). Development of a Deep Learning Algorithm for Periapical Disease Detection in Dental Radiographs. Diagnostics.

[8] von Wowern N. (2001). General and oral aspects of osteoporosis: A review. Clin. Oral Investig..

[9] Miller P.D. (2016). Management of severe osteoporosis. Expert Opin. Pharmacother..

[10] Gaetti-Jardim E.C., Santiago-Junior J.F., Goiato M.C., Pellizer E.P., Magro-Filho O., Jardim Junior E.G. (2011). Dental implants in patients with osteoporosis: A clinical reality?. J. Craniofacial Surg..

[11] Sukegawa S., Fujimura A., Taguchi A., Yamamoto N., Kitamura A., Goto R., Nakano K., Takabatake K., Kawai H., Nagatsuka H. (2022). Identification of osteoporosis using ensemble deep learning model with panoramic radiographs and clinical covariates. Sci. Rep..

[12] Alawi M., Begum A., Harraz M., Alawi H., Bamagos S., Yaghmour A., Hafiz L. (2021). Dual-Energy X-Ray Absorptiometry (DEXA) Scan Versus Computed Tomography for Bone Density Assessment. Cureus.

[13] Kermany D.S., Goldbaum M., Cai W., Valentim C.C.S., Liang H., Baxter S.L., McKeown A., Yang G., Wu X., Yan F. (2018). Identifying Medical Diagnoses and Treatable Diseases by Image-Based Deep Learning. Cell.

[14] Sangondimath G., Sen R.K., Rehman T.F. (2023). DEXA and Imaging in Osteoporosis. Indian J. Orthop..

[15] Singer A., McClung M.R., Tran O., Morrow C.D., Goldstein S., Kagan R., McDermott M., Yehoshua A. (2023). Treatment rates and healthcare costs of patients with fragility fracture by site of care: A real-world data analysis. Arch. Osteoporos..

[16] Rozylo-Kalinowska I. (2020). Panoramic Radiography in Dentistry. Imaging Tech. Dent. Radiol..

[17] Rondon R.H.N., Pereira Y.C.L., do Nascimento G.C. (2014). Common positioning errors in panoramic radiography: A review. Imaging Sci. Dent..

[18] Kaffe I., Fishel D., Gorsky M. (1977). Panoramic radiography in dentistry. Refuat Hapeh Vehashinayim.

[19] Perschbacher S. (2012). Interpretation of panoramic radiographs. Aust. Dent. J..

[20] Klemetti E., Kolmakov S., Heiskanen P., Vainio P., Lassila V. (1993). Panoramic mandibular index and bone mineral densities in postmenopausal women. Oral Surg. Oral Med. Oral Pathol..

[21] Singh Y., Atulkar V., Ren J., Yang J., Fan H., Latecki L.J., Ling H. Osteoporosis Prescreening and Bone Mineral Density Prediction using Dental Panoramic Radiographs. Proceedings of the Annual International Conference of the IEEE Engineering in Medicine and Biology Society.

[22] White S.C., Taguchi A., Kao D., Wu S., Service S.K., Yoon D., Suei Y., Nakamoto T., Tanimoto K. (2005). Clinical and panoramic predictors of femur bone mineral density. Osteoporos. Int..

[23] Halling A., Persson G.R., Berglund J., Johansson O., Renvert S. (2005). Comparison between the Klemetti index and heel DXA BMD measurements in the diagnosis of reduced skeletal bone mineral density in the elderly. Osteoporos. Int..

[24] Taguchi A., Tanaka R., Kakimoto N., Morimoto Y., Arai Y., Hayashi T., Kurabayashi T., Katsumata A., Asaumi J. (2021). Japanese Society for Oral and Maxillofacial Radiology Clinical guidelines for the application of panoramic radiographs in screening for osteoporosis. Oral Radiol..

[25] Kinalski M.A., Boscato N., Damian M.F. (2020). The accuracy of panoramic radiography as a screening of bone mineral density in women: A systematic review. Dentomaxillofac. Radiol..

[26] Calciolari E., Donos N., Park J.C., Petrie A., Mardas N. (2015). Panoramic Measures for Oral Bone Mass in Detecting Osteoporosis: A Systematic Review and Meta-Analysis. J. Dent. Res..

[27] Pallagatti S., Parnami P., Sheikh S., Gupta D. (2017). Efficacy of Panoramic Radiography in the Detection of Osteoporosis in Post-Menopausal Women When Compared to Dual Energy X-ray Absorptiometry. Open Dent. J..

[28] Scafoglieri A., Clarys J.P. (2018). Dual energy X-ray absorptiometry: Gold standard for muscle mass?. J. Cachexia Sarcopenia Muscle.

[29] Yeung A.W.K., Mozos I. (2020). The Innovative and Sustainable Use of Dental Panoramic Radiographs for the Detection of Osteoporosis. Int. J. Env. Res. Public Health.

[30] Lee J.S., Adhikari S., Liu L., Jeong H.G., Kim H., Yoon S.J. (2019). Osteoporosis detection in panoramic radiographs using a deep convolutional neural network-based computer-assisted diagnosis system: A preliminary study. Dentomaxillofac. Radiol..

[31] GitHub-open-mmlab/mmyolo: OpenMMLab YOLO Series Toolbox and Benchmark. Implemented RTMDet, RTMDet-Rotated, YOLOv5, YOLOv6, YOLOv7, YOLOv8,YOLOX, PPYOLOE, etc. https://github.com/open-mmlab/mmyolo.

[32] GitHub-ycwu1997/D-Persona: Official Code for Our CVPR 2024 (Highlight) Paper ‘Diversified and Personalized Multirater Medical Image Segmentation’. https://github.com/ycwu1997/D-Persona.

[33] COCO Dataset: All You Need to Know to Get Started. https://www.v7labs.com/blog/coco-dataset-guide.

[34] Releases Open-Mmlab/Mmpretrain. https://github.com/open-mmlab/mmpretrain/releases.

[35] ImageNet. https://www.image-net.org/.

[36] AdamW-PyTorch 2.3 Documentation. https://pytorch.org/docs/stable/generated/torch.optim.AdamW.html.

[37] Chou S.H., LeBoff M.S. (2017). Vertebral Imaging in the Diagnosis of Osteoporosis: A Clinician’s Perspective. Curr. Osteoporos. Rep..

[38] Vinayahalingam S., van Nistelrooij N., van Ginneken B., Bressem K., Tröltzsch D., Heiland M., Flügge T., Gaudin R. (2022). Detection of mandibular fractures on panoramic radiographs using deep learning. Sci. Rep..

